# Host–Microbe Interactions: Prospects of Machine Learning and Deep Learning Technologies in Animal Viral Disease Management

**DOI:** 10.3390/vetsci12121129

**Published:** 2025-11-27

**Authors:** Yiting Lu, Xiaowen Li, A. M. Abd El-Aty, Xianghong Ju, Yanhong Yong

**Affiliations:** 1 Marine Medical Research and Development Centre, Shenzhen Institute, Guangdong Ocean University, Shenzhen 518120, China; 2 College of Coastal Agricultural Sciences, Guangdong Ocean University, Zhanjiang 524088, China; 3 Zhanjiang City Key Laboratory of Animal Disease Prevention and Control Technology & Preparation Development, Zhanjiang 524032, China; 4 Department of Pharmacology, Faculty of Veterinary Medicine, Cairo University, Giza 12211, Egypt; 5 Department of Medical Pharmacology, Medical Faculty, Ataturk University, Erzurum 25240, Turkey

**Keywords:** machine learning, deep learning, animal viral diseases, interactions between host and microorganisms, animal growth monitoring

## Abstract

**Simple Summary:**

Viral diseases in farm animals spread quickly and can severely damage animal health and food production. At the heart of these infections is a complex “host–microorganism interaction”: viruses must enter animal cells, use the host’s machinery to multiply, and at the same time avoid being eliminated by the immune system. Understanding these interactions in a systematic way is difficult with traditional experiments alone because they are slow, expensive, and capture only part of this complexity. This review explains how modern computer methods, especially machine learning and deep learning, can help scientists study host–virus interactions more efficiently. We describe how these tools can analyze large sets of genetic and protein data to identify which animal molecules viruses use to enter cells and which host pathways they disrupt. We also summarize how computer models can support early detection of infection on farms and assist in selecting drug and vaccine targets that interfere with key host–virus interactions. Although challenges remain, such as limited data quality and rapidly changing viruses, this approach offers promising new ways to protect animal health and strengthen food security.

**Abstract:**

The rapid industrialization of global livestock production has intensified the threat of viral epidemics, in which the intestinal, respiratory, and reproductive systems are susceptible to viral attacks. Understanding the mechanism of virus–host interactions will facilitate the development of prevention strategies against highly mutable and fast-spreading pathogens. This review examines recent progress in applying machine learning (ML) and deep learning (DL) to the study and control of animal viral diseases. By analyzing existing research, we show how these techniques improve the prediction of host–microbe interactions, support continuous monitoring of animal health, and accelerate the discovery of drug targets and vaccine candidates. Integrating ML and DL frameworks enables more accurate modeling of complex biological processes and offers new tools for data-driven veterinary science. Nevertheless, challenges remain, including unbalanced datasets, the structural and evolutionary complexity of viruses, and the poor cross-species transferability of predictive models. Future work should emphasize algorithmic designs suited to small-sample, multivariate time series data and promote the development of intelligent systems that unite virology, immunology, and epidemiology. The combination of reinforcement learning for optimizing vaccination strategies and unsupervised learning for detecting emerging pathogens may ultimately lead to adaptive, efficient, and precise systems for disease prevention, supporting both animal health and sustainable livestock development.

## 1. Introduction

The prevention and control of animal infectious diseases constitute a cornerstone of modern livestock production and global public health security. With the accelerating pace of industrialization in animal husbandry, epidemic outbreaks have become more frequent and complex, characterized by rapid transmission, wide geographic dissemination, and high containment difficulty. Such dynamics have imposed substantial economic losses on the livestock industry and pose significant risks to food safety and zoonotic transmission. Although traditional preventive measures—including quarantine, vaccination, and biosecurity protocols—have played a critical role in disease mitigation, their effectiveness has been increasingly challenged by the rapid mutation of viral pathogens, the diversification of transmission routes, and the complexity of large-scale farming ecosystems [1,2].

Elucidating host–virus (or host–microbe) interactions is critical for the prevention and control of animal infectious diseases because it reveals the molecular determinants of host susceptibility and pathogen infectivity, identifies biomarkers suitable for early detection, and, via protein–protein interaction networks, uncovers key targets for vaccine or therapeutic intervention. Clarifying the mechanisms of receptor binding, immune evasion strategies, and stage-specific host responses facilitates the prioritization of surveillance targets and the design of species- and context-specific intervention strategies. Integrating these mechanistic insights with ML/DL-driven models helps bridge molecular discovery and population-level applications, thereby improving the interpretability and deployment readiness of predictive tools for animal disease prevention and control. Understanding viral–host protein interactions will help elucidate infection mechanisms and aid in the development of antiviral interventions. Viral infection is a multistage and dynamic process, with each stage characterized by distinct molecular mechanisms and each stage involving specific interaction networks whose temporal evolution determines the course of infection [3]. Traditional static analyses often overlook these dynamic aspects, whereas machine learning approaches—particularly recurrent neural networks (RNNs) and time series analysis techniques—can effectively capture temporal dependencies and nonlinear dynamics in molecular events [4]. By integrating multiomics datasets collected at different time points, researchers can construct time-resolved interaction networks, thereby providing a more comprehensive understanding of infection progression and host-response mechanisms [5].

Conventional pathogen research and surveillance have long relied on experimental methods such as viral isolation, serological testing, and molecular characterization. These approaches, while precise, are time-consuming, labor-intensive, and costly. Moreover, their capacity to capture dynamic host–virus interactions at the systems level is limited. In contrast, machine learning (ML) and deep learning (DL) technologies provide data-driven frameworks capable of learning complex, nonlinear relationships within large biological datasets, enabling the automated discovery of latent patterns that are often inaccessible through traditional methodologies [6]. In recent years, with the expansion of proteomics and transcriptomics, a vast amount of host–microbe interaction data has become available, offering new opportunities for computational modeling of infection dynamics and microbial ecology in animals. As proteomics and transcriptomics continue to expand, a wealth of host–virus interaction data has emerged, offering fertile ground for computational analysis. However, fully elucidating these intricate molecular networks via experimental means alone remains difficult [7,8]. ML and DL techniques can leverage such high-dimensional data to predict protein–protein interactions, infer mechanistic relationships, and identify potential therapeutic targets [9,10]. Furthermore, ML-based monitoring systems have shown increasing potential in tracking animal growth, behavior, and physiological responses, enabling early detection of infection-related anomalies and improving animal welfare and farm management efficiency.

In summary, the integration of machine learning and deep learning into veterinary virology has reshaped our ability to analyze and interpret complex biological systems. These computational approaches not only accelerate the identification of key molecular targets and vaccine candidates but also lay the foundation for intelligent, predictive, and adaptive systems in epidemic prevention and control. As artificial intelligence continues to advance, it promises to transform animal disease research from descriptive observation to proactive prediction, enabling the establishment of precision epidemiology and data-driven veterinary medicine in the near future.

## 2. Overview of Machine Learning and Deep Learning Technologies

To align with the scope of this review, this section narrows the overview of machine learning (ML) and deep learning (DL) to topics directly relevant to modeling animal viral diseases, using examples and a problem-oriented perspective to highlight method applicability and limitations. Machine learning refers to techniques that enable computers to learn from data and make predictions or decisions by constructing algorithms and models [11]. In veterinary virology, more interpretable classical models are commonly applied to rapid modeling of small-sample or structured surveillance data (for example, sequence-based lineage classification or sensor-based detection of mastitis/behavioral anomalies), yet they often face clear challenges when confronted with class imbalance, longitudinal farm time series, and cross-species transfer. ML typically employs relatively simple algorithms—such as random forests and support vector machines—which perform well on modestly sized datasets and are generally easier to interpret [12]. Random forests, in particular, leverage ensembles of decision trees to provide robust feature selection and noise tolerance, thereby enabling effective integration of heterogeneous data sources such as sequence conservation, domain annotations, and Gene Ontology terms [13]. In several applied settings, random forests have been used to fuse sparsely labeled genomic and phenotypic data to enhance predictive robustness. Support vector machines excel in high-dimensional feature spaces: their kernel functions enable flexible modeling of complex nonlinear relationships, including protein structural properties, physicochemical attributes, and evolutionary conservation [14].

Deep learning, a subfield of ML, focuses on constructing and training multilayer neural networks that can automatically learn complex patterns from large datasets. Its adoption has transformed protein–protein interaction prediction. Convolutional neural networks (CNNs), through hierarchical feature extraction and weight sharing, are effective at identifying intricate motifs in biological data and have been successfully applied to tasks such as sequence motif discovery, protein function prediction, and molecular design [15]. Nevertheless, DL’s dependence on large, high-quality labeled datasets poses a distinct limitation in veterinary contexts, and issues of cross-species generalization and model interpretability remain pressing. Long short-term memory (LSTM) networks, with built-in memory cells, overcome the shortcomings of traditional approaches for long-range sequence dependencies and enable the extraction of deep semantic features from protein sequences [16]. For farm-level longitudinal monitoring (e.g., behavioral and physiological time series), LSTM and other temporal models show promise but typically require domain-adaptation or transfer-learning strategies to address device heterogeneity, environmental drift, and temporal evolution. The introduction of attention mechanisms has further advanced capabilities: cross-attention architectures dynamically reweight inputs to focus the model on sequence regions most relevant to interaction prediction, improving both predictive accuracy and interpretability [17]. Addressing class imbalance, few-shot learning, external validation, and standardized benchmarking is particularly critical for deploying these methods in animal virology. Compared with traditional ML approaches, deep learning offers stronger automatic feature-learning abilities and is better suited to large-scale, high-dimensional datasets [18].

**Figure 1:**
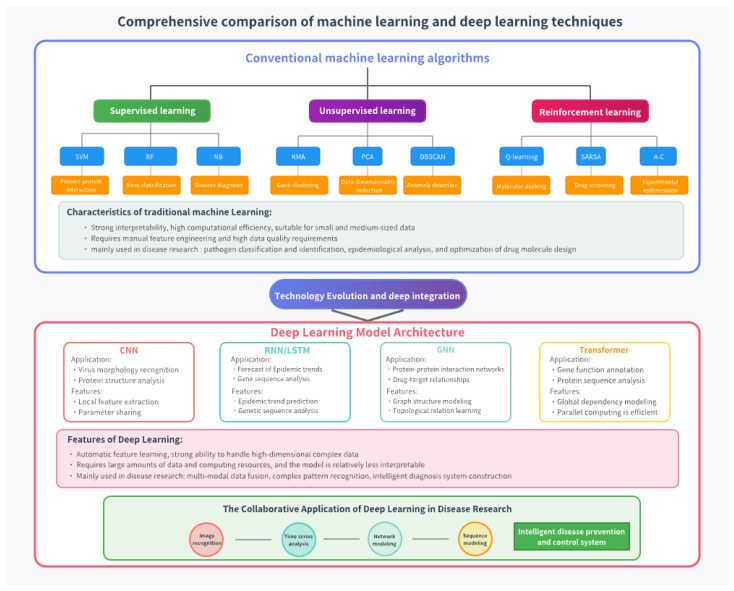
Comprehensive comparison of machine learning and deep learning technologies.

Figure 1 provides a systematic overview comparing machine learning and deep learning techniques, particularly in the context of disease research and biomedical data analysis.

At the top, the figure distinguishes conventional machine learning algorithms into three main categories: supervised learning, unsupervised learning, and reinforcement learning. Each branch lists representative algorithms (e.g., SVM, R F, K-means, and Q-learning) along with their specific applications, such as protein–protein interaction prediction, gene clustering, and drug screening. Traditional methods are described as computationally efficient and interpretable but are limited by their dependence on manual feature engineering and smaller datasets. The middle section, labeled Technology Evolution and Deep Integration, transitions into the deep learning era. This section highlights four core architectures—CNN, RNN/LSTM, GNN, and transformer—and outlines their application scopes (e.g., virus morphology recognition, epidemic trend forecasting, protein network modeling, and gene function annotation). Each model type is accompanied by its distinct functional features, such as local feature extraction for CNNs or topological relation learning for GNNs. Deep learning is characterized by its ability to automatically extract complex high-dimensional features, albeit at the cost of interpretability and increased data requirements. Finally, the figure summarizes the collaborative applications of deep learning in disease research, encompassing image recognition, time series analysis, network and sequence modeling, and intelligent disease prevention systems. Together, these modules illustrate the progressive integration of machine learning and deep learning toward constructing data-driven, intelligent medical diagnostic frameworks. The figure was created via js.design.

## 3. Surveillance and Diagnostics

Early detection and accurate characterization of viral infections in animals depend on surveillance systems capable of handling both genomic and phenotypic complexity. Machine learning (ML) and deep learning (DL) methods have now been embedded into multiple components of this pipeline, from sequence-based classification to sensor- and image-based monitoring, and are gradually moving from purely in silico validation to on-farm evaluation under real production conditions.

### 3.1. Sequence-Based Surveillance

In porcine reproductive and respiratory syndrome virus (PRRSV), fine-scale lineage classification has long been a major challenge in veterinary virology. Traditional classification approaches rely on manual sequence alignment and phylogenetic tree reconstruction, which are labor intensive and prone to subjective bias when dealing with large-scale sequence datasets. With the rapid expansion of high-throughput sequencing and the exponential growth of viral genomic data, there is an urgent need for more efficient, objective, and fully automated classification tools [19] in Table 1 and Table 2.

Kim et al. used ORF5 amino acid sequences and derived physicochemical properties such as Atchley factors to construct feature vectors and trained multiple ML classifiers, including random forests, to learn discriminative sequence motifs within the existing PRRSV-2 sublineage/lineage framework. These models achieved >95% accuracy in automatically assigning newly generated ORF5 sequences to predefined lineage categories, thereby algorithmically replacing the traditional phylogeny-based typing workflow with an automated alternative [20].

In contrast, VanderWaal et al. did not focus solely on “faster classification” within a fixed framework. Instead, using more than one hundred thousand PRRSV-2 ORF5 sequences, they applied phylogenetic clustering combined with genetic distance thresholds to introduce a finer-grained “genetic variant” level beneath the classical lineage/sublineage scheme. This resulted in a dynamically updatable nomenclature system and was coupled with ML models and online tools to automatically map newly sequenced strains to established variants [21].

Sequence-based surveillance has also advanced considerably for avian influenza virus (AIV). Because the AIV genome is segmented and its RNA polymerase lacks proofreading activity, point mutations and reassortment occur frequently [22,23], making it particularly difficult to infer phenotypes from sequences alone. Using deep learning, researchers have analyzed the predicted three-dimensional structures of the hemagglutinin protein in combination with principal component analysis for dimensionality reduction and anomaly detection algorithms. When trained on 12,143 AIV genomes, the resulting model accurately distinguished highly pathogenic H5 and H7 subtypes from low-pathogenicity strains with an accuracy exceeding 0.9, providing a key intelligent tool for the rapid identification of global AIV threats [24]. Such models can automatically generate “pathogenicity labels” for newly sequenced strains and can be seamlessly integrated into routine sequence analysis workflows.

For Newcastle disease virus (NDV), investigators have developed several ML models, including random forests and support vector machines. Using nucleotide and amino acid features derived from F and HN protein genes as input variables, these models can replace traditional, time-consuming hemagglutination inhibition assays, enabling rapid and precise prediction of antigenic distances among strains and providing an efficient computational tool to support vaccine strain selection and immunization strategy design [25]. Overall, these studies illustrate how ML-enabled sequence analysis can transform viral genomes from passively stored data into high-value resources for active surveillance.

### 3.2. Sensor- and Image-Based Monitoring

In addition to molecular information, modern livestock operations continuously generate large volumes of audio, image, and thermal imaging data that reflect behavioral and physiological changes associated with infection. Deep learning models are increasingly used to mine these signals to achieve noninvasive, early warning surveillance.

In poultry production, researchers have developed one-dimensional convolutional neural networks (1D-CNNs) incorporating batch normalization and max pooling layers to extract deep features from vocalization data, enabling automatic classification of flock health status on the basis of audio recordings [26]. In addition, Hassan et al. introduced a custom “burn layer” that applies random perturbations to the input during training to increase robustness against environmental noise and varying recording conditions. Combined with global average pooling and the Adamax optimizer, this framework can accurately discriminate between healthy and diseased flocks via sound signals alone [27].

Visual modalities provide complementary information. Multilayer convolutional neural networks have been trained to analyze images of poultry feces and differentiate normal from pathological conditions, including coccidiosis, salmonellosis, and Newcastle disease [28]. Under real farm conditions, a fine-tuned MobileNetV2 model achieved an accuracy of 98.24%, demonstrating that such models can function as low-cost, onsite screening tools that offer a more economical and convenient intelligent alternative to conventional PCR-based diagnostics [29].

Thermal imaging combined with ML has likewise been applied to detect temperature-related signatures of early infection. By extracting features from infrared thermograms and feeding them into support vector machines and artificial neural networks, researchers achieved early diagnostic accuracies of 97.2–100% in flocks infected with avian influenza or Newcastle disease, with abnormalities detectable as early as 24 h post-infection [30]. These noncontact, flock-level monitoring systems are particularly well suited for precision livestock farming and are highly important for continuous, low-disturbance health surveillance.

However, several biological and practical limitations must be recognized. First, the quality of on-farm audio, image, and thermal data is highly sensitive to sensor placement, occlusion, illumination changes, and device aging, and the monitoring unit is usually a flock rather than individual animals. This makes it difficult to precisely link signals to individual infection status and introduces noise and labeling errors. Second, mixed infections, changes in stocking density, fluctuations in ventilation and temperature–humidity conditions, and noninfectious stressors related to feed and management can all alter behavior and surface temperature patterns. These factors overlap with viral infection signatures and act as important confounders, increasing the risk of misclassification when models are deployed in real-world settings. Third, most existing models are trained under relatively controlled experimental conditions, and cross-farm, cross-season, and cross-production system validation remains limited; thus, their generalizability and long-term stability require systematic evaluation.

Consequently, audio-, image-, and thermal imaging-based intelligent monitoring tools are better suited as front-end early warning and risk stratification instruments. They can rapidly flag “suspicious barns” or “abnormal flocks” in large-scale operations, thereby optimizing sampling strategies and improving surveillance efficiency rather than replacing nucleic acid tests, serology, or virus isolation. Only when these models are consistently cross-validated against laboratory diagnostic results and continuously recalibrated through standardized annotation workflows and quality control can they be expected to play a stable and reliable role in practical surveillance systems for avian influenza and Newcastle disease.

## 4. Epidemiology and Risk Modeling

Epidemiological and risk models shift the focus from individual animals or single farms to entire production systems and regional scales. By integrating observational data with ML algorithms, these models aim to predict epidemic dynamics, quantify farm-level risk, and support the prioritization of limited control resources.

### 4.1. Temporal and Spatial Forecasting

Foot-and-mouth disease virus (FMDV) exhibits extremely high genetic variability, and the receptor-binding regions of its VP1 protein frequently accumulate point mutations, resulting in substantial differences in receptor affinity among strains. In addition, FMDV can exploit multiple cell-surface receptors, including integrins αvβ3, αvβ6, αvβ8 and heparan sulfate proteoglycans [73], which complicates epidemic prediction. This plasticity in receptor usage affects the host range and transmission characteristics and makes traditional biochemical and epidemiological analyses more challenging. Therefore, ML-based prediction frameworks have been explored as practical and cost-effective tools for early FMD warning.

Using farm-level data, Punyapornwithaya et al. applied classification trees, random forests, and chi-square automatic interaction detection (CHAID) to predict FMD outbreaks, achieving accuracies ranging from acceptable to excellent and demonstrating that ML methods can be effectively integrated into existing surveillance systems to strengthen their predictive ability [31]. Kapalaga et al. further optimized random forest performance to address degradation in predictive accuracy under varying data distributions and compared multiple ML models, including support vector machines, logistic regression, and gradient boosting machines, thereby establishing a more robust and reliable framework for FMD epidemic prediction [32].

### 4.2. Farm-Level Risk Scoring and Biosecurity Drivers

Farm-level risk models synthesize complex management and environmental information into quantitative scores that reflect the likelihood of disease introduction or persistence. For PRRSV, researchers have used ML algorithms to process large datasets containing 260 biosecurity variables, employing supervised learning methods such as random forests to automatically identify and rank key biosecurity practices associated with PRRS outbreaks. These models achieved 76–80% accuracy in predicting farm-level disease risk [33], providing data-driven support for revising biosecurity guidelines and designing targeted training and extension programs.

Similar strategies have been applied in animal disease control and health monitoring programs, where ML models combine farm demographics, animal movement records, vaccination status, and local epidemic conditions to assign risk categories or generate prioritized inspection lists. For example, Machado et al. trained a random forest model using cross-sectional epidemiological data from Brazilian dairy farms and, through variable importance analysis, identified several key biosecurity factors—such as the type of insemination personnel, number of neighboring cattle farms, and routine use of rectal examination—that were strongly associated with bovine viral diarrhea virus (BVDV) positivity [34]. These findings showed that ML can be used to quantitatively assess infection risk at the farm level and provide concrete targets for improving reproductive management and regional biosecurity strategies. Ferri et al. used historical field inspection data to construct a model that outputs a risk-ranked “priority farm list” to guide risk-based onsite animal welfare inspections [35]. Such risk scoring systems can be embedded into routine surveillance frameworks to direct inspectors toward high-risk premises, thereby improving the probability of detecting subclinical infection or biosecurity breaches under resource constraints.

The practical value of these models depends not only on predictive accuracy but also on interpretability. Translating model outputs into actionable recommendations for biosecurity improvement is a critical step toward achieving real-world impact. Moreover, these models are typically derived from data collected in specific regions and time periods, and training datasets may suffer from issues such as low numbers of positive cases and incomplete case reporting. As a result, they are inevitably influenced by data bias and temporal drift. Without repeated validation in other regions or production systems, their performance and stability in broader contexts remain uncertain. Furthermore, some risk factors identified as “high importance” may be costly or difficult to modify in practice, so balancing statistical significance, economic feasibility, and managerial operability will be an important consideration for future implementation.

## 5. Host–Pathogen Molecular Interactions and Mechanistic Inference

### 5.1. Protein Function and Structure Inference

The African swine fever virus (ASFV) genome contains many open reading frames (ORFs), especially within multigene family regions whose functions have long remained unclear. Traditional homology-based methods are often ineffective because of the lack of closely related sequences [36]. To address this, researchers have developed ML frameworks specifically tailored to viral proteins, typically adopting multitask learning architectures that use sequence-derived features to predict multiple functional attributes simultaneously, such as subcellular localization, family membership, and enzymatic activity [37]. By exploiting correlations among different functional properties, these models substantially improve annotation accuracy and have been applied to identify putative virulence-associated genes when the genomes of ASFV strains with differing pathogenicity are compared [38].

ML has also been used to analyze virus–host protein interaction networks. In ASFV, topological analysis of inferred virus–host interaction graphs has enabled the identification of critical viral targets and host factors, providing mechanistic hypotheses for how ASFV rewires host pathways during infection [39]. Although some studies have extended these networks to in silico drug screening, the interaction maps themselves already offer an important structural framework for explaining differences in virulence and tissue tropism.

At the structural level, the advent of the AlphaFold system has greatly expanded opportunities for mechanistic inference in ASFV research. By integrating evolutionary information, physicochemical constraints, and known structural data through deep learning, AlphaFold can predict three-dimensional protein structures with near-experimental accuracy, thereby compensating for the limitations of X-ray crystallography and cryo-EM for certain ASFV proteins [40]. For example, high-confidence structural predictions for the ASFV CD2v protein have enabled researchers to refine its domain architecture and precisely localize key amino acid residues responsible for erythrocyte binding, providing a structural explanation for hemadsorption and its relationship with virulence [41]. Similarly, structure prediction for 193 proteins encoded by the ASFV Georgia 2007 strain has markedly expanded the structural coverage of the ASFV proteome, furnishing a rich resource for subsequent mechanistic studies [42].

### 5.2. A General Method for Predicting Protein Interactions Between Hosts and Microorganisms

Computational strategies for predicting protein–protein interactions (PPIs) have evolved from traditional statistical learning methods to advanced deep neural architectures. Early ML approaches demonstrated notable predictive capabilities: Chen et al. utilized random forest algorithms that combine sequence conservation, domain information, and gene ontology annotations to achieve strong performance on human PPI datasets [43]. Similarly, Aherfi et al. applied support vector machine (SVM) models that integrated spatial structure, physicochemical, and evolutionary features to identify key viral–host interaction pairs in coronavirus infections [44].

With the rise of DL, neural network-based approaches have become dominant. Zhang et al. developed the CBIL-VHPLI framework, which combines bidirectional LSTMs with CNNs for predicting interactions between viral proteins and host long noncoding RNAs (lncRNAs) [45]. Madan et al. leveraged RNN variants to learn deep sequential dependencies within viral and host protein sequences, increasing the accuracy of interaction prediction [46]. GNN-based models have also emerged as powerful tools: Koca et al. used semisupervised GCNs to leverage both labeled and unlabeled data for novel virus–human interaction prediction [47], whereas Baranwal et al. designed the Struct2Graph architecture using graph attention networks to infer PPIs directly from 3D structural data with a prediction accuracy of 98.89% [48].

Further improvements have been achieved through attention mechanisms. Tsukiyama et al. combined cross-attention modules with one-dimensional CNNs in their PHV predictor, which is capable of processing protein sequences of up to 9000 amino acids and generating high-quality feature representations for virus–host PPI prediction [49]. These developments collectively highlight the transformative role of DL in enabling accurate, interpretable, and scalable modeling of biological interaction networks.

**Figure 2:**
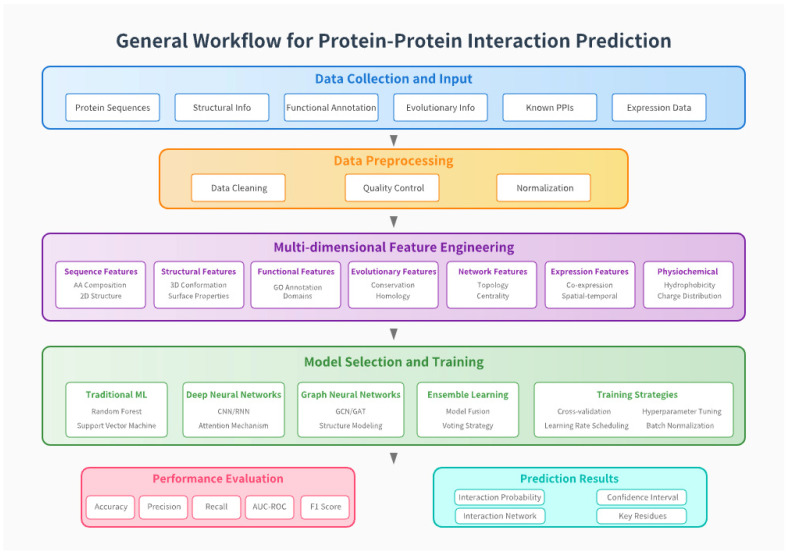
General workflow of protein–protein interaction prediction.

Figure 2 illustrates the general workflow for protein–protein interaction (PPI) prediction. The process begins with data collection, integrating protein sequences, structural information, functional annotation, evolutionary data, known PPIs, and expression profiles. After data preprocessing, including cleaning, quality control, and normalization, multidimensional feature engineering extracts sequence, structural, functional, evolutionary, network, and physicochemical characteristics. Subsequently, various machine learning models—such as traditional algorithms, deep and graph neural networks, and ensemble approaches—have been applied and optimized through training strategies. Finally, model performance is evaluated via metrics such as accuracy, precision, and AUC-ROC, yielding predictions of interaction probability, key residues, and confidence intervals.

## 6. Interdisciplinary Integration and Emerging Cross-Domain Technologies

Viral infection is a multifactorial process involving genomic alterations, protein interactions, immune responses, and ecological influences. These complexities indicate that future disease prevention systems must rely on interdisciplinary integration and cross-domain technological innovation. In this context, ML and DL are not merely analytical tools but serve as crucial bridges linking biological sciences, computational modeling, and engineering technologies.

For example, the integration of multiomics analysis with artificial intelligence (AI) is accelerating the systematic elucidation of virus–host interaction mechanisms. Network pharmacology and protein interaction prediction are reshaping antiviral drug discovery paradigms, whereas digital twin and Internet of Things (IoT) technologies enable real-time monitoring and dynamic simulation of disease processes, shifting epidemic management from passive response to proactive prediction and intervention. Concurrently, the application of intelligent algorithms in vaccine design and immunological modeling is shortening R&D cycles and improving antigenic specificity. Together, these emerging directions enrich methodological diversity and lay a strong foundation for the development of intelligent and sustainable animal disease prevention systems.

### 6.1. Multiomics Integration and Artificial Intelligence

With the rapid advancement of high-throughput sequencing and omics technologies, research on animal viral diseases has evolved from single-gene or protein studies to system-level biological analyses that integrate genomics, transcriptomics, proteomics, and metabolomics. However, the high dimensionality, noise, and heterogeneity of multiomics data pose challenges for traditional statistical methods, which often fail to capture the underlying biological patterns. ML and DL provide new opportunities for the integrative analysis of complex, high-dimensional datasets [50,51].

In supervised ML, algorithms such as support vector machines (SVMs) and random forests (RFs) are frequently used to identify virus-associated biomarkers from large-scale omics features and to distinguish between healthy and infected samples. Ensemble methods such as extreme gradient boosting (XGBoost) demonstrate strong robustness in handling nonlinear relationships and small-sample datasets, as shown in predictive modeling of H9N2 avian influenza using farm-level production, health, and environmental monitoring data [52]. In contrast, unsupervised learning methods, including principal component analysis (PCA) and K-means clustering, can reveal hidden structures in unlabeled data, such as strain differentiation or host susceptibility subgroups. Additionally, multitask learning models have been introduced to perform simultaneous disease classification, progression prediction, and pathway discovery, thereby providing an integrated analytical framework for multi-omics studies [53].

DL plays an even more prominent role in multiomics integration. Convolutional neural networks (CNNs) efficiently extract local patterns in feature matrices, whereas recurrent neural networks (RNNs) and long short-term memory (LSTM) architectures capture temporal variations in transcriptomic data to model infection dynamics. Autoencoders (AEs) and variational autoencoders (VAEs) perform feature compression and denoising while retaining essential biological information. For example, Wang et al. proposed the FactVAE framework to achieve effective integration of single-cell multiomics data [54], whereas Li et al. used an improved deconfounding VAE to precisely define disease subtypes [55]. Graph neural networks (GNNs), which model topological relationships among proteins or genes, have become essential tools for predicting virus–host protein interactions (PPIs). By learning the latent dependencies in biological networks, GNN-based models can identify key regulatory nodes and potential therapeutic targets. Koca et al. built a graph convolutional network (GCN) model to predict virus–human PPIs with high accuracy and stability [47]. Similarly, Jiang et al. integrated pretrained protein language models with graph attention networks (GATs) to enhance cross-virus generalization in interaction prediction [74].

With respect to animal host–virus interactions, several empirical multiomics plus AI case studies have emerged in recent years. For example, in porcine reproductive and respiratory syndrome (PRRS), Zhang et al. integrated metabolomic and lipidomic data from serum samples of clinical pig herds and, in combination with machine learning, distinguished infected from noninfected animals, identified lysophosphatidic acid as a key metabolite closely associated with infection outcome, and further confirmed its role as a diagnostic biomarker and potential therapeutic target, thereby providing multiomics evidence linking molecular phenotypes to clinical endpoints [56] (Table 2). In bovine respiratory disease (BRD), Li et al. combined genomic, transcriptomic and metabolomic datasets from feedlot cattle and applied several supervised learning algorithms to screen candidate loci and metabolic pathways associated with infection susceptibility, demonstrating the potential of a multiomics–machine learning framework for dissecting the relationship between complex host genetic backgrounds and disease risk [57]. In a swine influenza model, joint transcriptomic and proteomic analyses of upper and lower airway tissues, together with downstream clustering and pathway enrichment, have been used to characterize differences in signaling pathways and innate immune responses induced by distinct viral strains at early stages of infection, laying the foundation for subsequent machine learning-based selection of informative pathways and marker genes [58].

Overall, these methods enable a multilevel understanding of viral infection mechanisms. Through feature selection, pattern recognition, and dynamic modeling, ML and DL methods reveal regulatory networks, predict disease progression, and support precision intervention [59]. As large-scale, high-quality multiomics databases expand and model interpretability improves, the deep integration of AI and multiomics will likely become a central research direction in animal virology, driving the development of intelligent, data-driven disease surveillance systems.

### 6.2. Integration of Network Pharmacology with Machine Learning

Network pharmacology, as a systematic approach to drug discovery, offers a new paradigm for understanding and developing therapeutics against animal viral diseases. Unlike traditional single-target strategies, viral infections involve multiple molecular pathways, often rendering single-target drugs insufficient for achieving effective outcomes. In this context, the integration of machine learning (ML) with network pharmacology provides unique advantages by simultaneously considering the synergistic effects of drugs on multiple targets, thereby enabling more accurate prediction of overall pharmacological responses [60].

For example, in the development of ASFV inhibitors, the combination of ML models with molecular docking techniques has significantly improved the efficiency of drug candidate screening. By analyzing large-scale compound–target affinity datasets, ML algorithms can rapidly identify and prioritize compounds with high biological activity [61,62]. In the search for ASFV polymerase inhibitors, ML-based prediction frameworks have proven particularly valuable for analyzing massive compound libraries, successfully identifying multiple potential molecules that were subsequently validated through in vitro experiments for strong inhibitory activity against viral polymerase [63,64]. These studies demonstrate the critical role of ML in accelerating antiviral drug discovery.

From a technical perspective, deep learning (DL)-based architectures such as graph convolutional networks (GCNs) and variational autoencoders (VAEs) have enabled high-dimensional molecular feature representation and precise prediction of drug–target interactions [65]. In parallel, traditional ML methods such as random forests (RFs) and support vector machines (SVMs) continue to play key roles in mining multidimensional clinical datasets, contributing to the construction of intelligent veterinary drug decision-support systems for drug discovery, dosage optimization, and adverse effect prediction [75]. Beyond these classical approaches, deep learning architectures have also been successfully applied to veterinary datasets. For example, Zhu et al. employed a DL-enhanced protein–protein interaction modeling strategy to predict antiviral compounds targeting African swine fever virus (ASFV), demonstrating that deep neural feature extraction can effectively guide drug candidate prioritization in a major livestock disease system [39]. This case highlights how DL architectures originally developed for human biomedical applications can be adapted to support drug discovery efforts in veterinary medicine.

Furthermore, ML and DL techniques have been utilized to analyze microRNA–mRNA regulatory networks, facilitating the identification of novel therapeutic targets and the prediction of drug–target interaction mechanisms. By integrating gene expression profiles and biomarker data from individual animals, these models can be used to construct personalized therapeutic response prediction systems, forming a closed-loop process that connects mechanistic understanding with clinical application. Collectively, such AI-driven frameworks enable a comprehensive, intelligent pipeline for veterinary drug R&D, encompassing molecular mechanism elucidation, candidate identification, and optimized clinical application [76].

Importantly, although the combination of network pharmacology and machine learning offers clear advantages in candidate compound prioritization and in generating hypotheses for multitarget mechanisms, multiple practical constraints remain at the level of clinical application in animals. First, from a pharmacological standpoint, multitarget interventions inherently entail a greater risk of off-target effects and more intricate pharmacodynamic interactions: within highly interconnected signaling networks, the simultaneous modulation of several nodes may yield synergistic benefits but may also provoke more severe adverse reactions [77]. Second, pharmacokinetic (ADME), tissue distribution, and host-specific toxicological datasets for food-producing animals have long been relatively scarce, while the interspecies variability in ADME characteristics is substantial and often difficult to predict, making it unreliable for ensuring dosing safety and efficacy through simple cross-species extrapolation [78]. In addition, in the domain of food-producing animals, strict regulatory obligations—such as maximum residue limits (MRLs) and withdrawal periods—must also be fulfilled, which objectively raises the entry threshold for introducing novel antiviral agents into veterinary practice [79]. Taken together, a more prudent position at present is to regard “network pharmacology plus machine learning” as an upstream toolkit that can shorten the drug development timeline and broaden the pool of candidates rather than a mature technology already capable of supporting large-scale field medication.

### 6.3. Digital Twin and Intelligent Monitoring

In livestock production, digital twin systems have become a key technology for real-time monitoring and dynamic updating. By constructing virtual models that mirror physical entities (e.g., animal populations, farm environments, or production systems), digital twins have emerged as essential tools for precision agriculture and intelligent management. In animal husbandry, they not only reflect health status in real time but also support environmental surveillance, disease prediction, and resource optimization across multiple dimensions [80]. Typically, a digital twin system comprises perception, network, and application layers that work in concert to enable continuous monitoring and predictive analytics of animal health and environmental conditions [81].

The convergence of edge computing and deep learning has substantially improved processing efficiency by reducing latency and bandwidth consumption. In particular, for livestock health monitoring, edge computing enables near-field preprocessing and analysis, allowing rapid detection of anomalies and timely alerts [82]. This distributed processing paradigm not only enhances real-time responsiveness but also reduces the computational load on the cloud. For example, in some poultry farms, sensors continuously track behavioral patterns, body temperature, and environmental parameters such as humidity. Combined with DL-based analytics, these data streams enable accurate identification of emerging health issues or environmental stressors [83]. Furthermore, integration with the Internet of Things (IoT) provides seamless connectivity for environmental monitoring and livestock management, allowing producers to track health trends and environmental changes in real time and to implement early interventions when disease risk is detected [84].

With ongoing technological advances, the application prospects of digital twins and intelligent monitoring in animal husbandry are expanding. For example, digital epidemiology leverages digital twins and IoT infrastructures to simulate transmission pathways, thereby aiding in the prediction and control of outbreaks [85]. As digital twin systems continue to evolve—particularly through the fusion of DL and multisource data integration—they are expected to improve management efficiency and provide more precise decision support for disease prevention, resource allocation, and environmental protection.

In summary, the adoption of digital twins in livestock systems—especially their integration with low-power networks, edge computing, and DL—has already driven transformative changes in the industry. Continued development of this technology stack is anticipated to promote animal husbandry toward a more intelligent, precise, and sustainable trajector.

### 6.4. Intelligent Assistance for Vaccine Design

As one of the core objectives in animal disease prevention, vaccine design has increasingly benefited from the integration of machine learning (ML) and deep learning (DL) technologies. These approaches enable large-scale analysis of genomic and proteomic datasets, allowing precise identification of antigen candidates with high immunogenic potential [86].

In reverse vaccinology (RV), ML and DL play critical roles in the predictive identification of viable vaccine components. The application of these methods has significantly improved the accuracy of antigen prediction on the basis of protein sequence features, overcoming the limitations of traditional filtering methods [29]. For example, the Vaxign-DL framework employs a three-layer fully connected neural network to compute 509 biological and biomedical feature parameters, achieving high-precision prediction of protective bacterial antigens. The model demonstrated excellent performance, with an area under the ROC curve (AUC) of 0.94, specificity of 0.99, sensitivity of 0.74, and overall accuracy of 0.96 [66] in Table 1 and Table 2.

For epitope identification, ML and DL algorithms analyze large volumes of published experimental data to detect sequence regions with epitope-like properties [67]. This process integrates diverse data sources—including peptide arrays, cryo-electron microscopy reconstructions, and protein structure predictions—and applies classical models such as random forest (RF) and support vector machines (SVMs) for the precise prediction of both linear and conformational epitopes [68]. These approaches not only reduce the number of experimental candidates required for validation but also substantially accelerate the design cycle of epitope-based vaccines.

In practical applications, Venkateswaran et al. developed a comprehensive computational platform that combines the BepiPred-2.0 server (RF-based B-cell epitope prediction), the AlphaFold3 system (DL-based 3D protein structure modeling), and the C-ImmSim simulator for immune response prediction, collectively supporting the design of multiepitope ASFV vaccines [69]. Recent advances in ML-assisted epitope mapping have further strengthened the accuracy of epitope identification and antigen prioritization [70]. Similarly, in SARS-CoV-2 research, DL-based deep neural network (DNN) architectures have replaced traditional multistep prediction workflows, directly analyzing spike protein sequences to predict 26 potential vaccine subunits [71].

Overall, the integration of ML and DL into vaccine design provides a powerful computational foundation for rational antigen discovery. By combining predictive modeling, structural analysis, and immunological simulation, AI-driven vaccine design significantly enhances efficiency and precision, paving the way for next-generation vaccines in veterinary medicine.

## 7. Limitations and Challenges of Machine Learning and Deep Learning

Machine learning (ML) and deep learning (DL) have become indispensable tools in biological research, particularly in protein interaction prediction, drug development, and vaccine design. Despite their remarkable progress and potential, several technical and practical challenges still constrain their broader application in the study of animal viral diseases.

### 7.1. Technical Challenges

In the context of animal viral disease research, data quality remains one of the most critical issues affecting ML and DL model performance. Biological datasets are often noisy, incomplete, and inconsistently labeled, leading to biased or unreliable model outputs. For example, in predicting the functions of African swine fever virus (ASFV) proteins, the lack of comprehensive structural data limits the model’s ability to capture the full biological context, thereby reducing prediction accuracy.

Moreover, inconsistencies in data quality and standardization across research groups and experimental methodologies hinder effective data integration and comparative analysis [87]. Owing to the limited availability of experimental samples in many animal viral studies, DL models are prone to overfitting, learning dataset-specific noise rather than generalizable biological patterns. Overfitting not only compromises reliability but also weakens predictive performance on unseen data [51]. In the case of deep multimodal models used for Newcastle disease diagnosis, models may become overly dependent on specific modalities, resulting in poor adaptability to new or mutated viruses.

Another significant challenge is data imbalance, such as unequal proportions of healthy and infected animal data, which skews model predictions toward majority classes and undermines overall accuracy 7. The well-known “black box” problem of DL models further limits their interpretability. Although DL algorithms effectively learn complex nonlinear relationships from large datasets, their decision-making processes often lack transparency [88]. In viral epidemiology, for example, ML models may accurately predict an FMD outbreak but fail to provide biologically interpretable explanations for why those predictions were made, reducing scientific trust and diagnostic usability.

From the perspective of cross-species generalizability, many current universal protein language models, network pharmacology pipelines, and interaction predictors are trained primarily on data from humans, mice, and other model organisms and are then “transferred” for use in analyzing virus–host interactions or screening candidate targets in livestock species such as pigs, cattle, or poultry. However, comparative genomics and enhancer prediction studies have demonstrated that machine learning models trained on human or mouse data often exhibit a significant decline in predictive performance when applied to nonmodel livestock species, such as cattle and pigs. This indicates that additional species-specific data are required for retraining or fine-tuning the models [89]. Consequently, in protein function prediction and the identification of host resistance factors, when training data mainly originate from human or model-virus systems, the output “candidate targets” may suffer from systematic biases or even fail entirely in true food-producing animals.

Additionally, DL models require substantial computational resources and interdisciplinary collaboration. The training and inference of complex neural networks, particularly those involving genomic or multiomics data, demand significant computational power and memory resources [90]. Although cloud computing and high-performance computing platforms have alleviated some constraints, high costs and long training times still pose barriers to widespread adoption.

### 7.2. Application Challenges

The rapid mutation of animal viruses poses a significant challenge for applying machine learning and deep learning technologies in prediction and surveillance. Viral mutations can lead to changes in the genome, structure, and pathogenicity, causing an inevitable domain shift and temporal drift between the data distribution encountered during the training phase and the true distribution during the deployment phase [91]. In the case of African swine fever virus (ASFV), the high mutation rate makes it difficult for existing deep learning models to accurately predict mutation trends, thus affecting the reliability of the prediction results. Similarly, in machine learning studies on highly pathogenic avian influenza viruses (such as H5 subtypes) and human influenza, previous work has shown that static models trained only once struggle to address long-term spatiotemporal risk variations. To maintain reasonable predictive performance in real-world monitoring scenarios, dynamic updates and reevaluations using multiyear and multiregional data are needed [92].

Another challenge lies in the difficulty of data labeling. Generating reliable labeled data for biological systems requires costly experimental design and specialized expertise. In the face of rapidly mutating viruses, new variants may emerge faster than the corresponding annotated data can be produced, compromising the timeliness and effectiveness of model training [93]. Ethical and privacy considerations also arise when using animal data in ML research. In many regions, animal experimentation and data collection are strictly regulated by ethical review processes, which can limit data availability and utilization [94]. Moreover, safeguarding data privacy and preventing misuse have become essential components of responsible AI deployment in biological research. As AI technologies continue to expand within the life sciences, balancing innovation with ethical responsibility will be a critical challenge moving forward. Furthermore, in machine learning research on animal viral diseases, external validation and prospective evaluation in real farm environments are insufficient. Many models are still confined to internal cross-validation within single laboratory datasets or single-scenario data and lack systematic “external testing” across regions, farms, or poultry facilities. In the case of bovine mastitis and other livestock health scenarios, studies have utilized multifarm automated milking system data for machine learning modeling and performed external validation on independent farms. The results revealed that predictive performance varied significantly among farms, highlighting the substantial impact that farm-specific factors have on model generalizability [95]. Further research suggests that without “customized” calibrations that incorporate specific farm management practices and health levels, a unified model is unlikely to maintain stable performance across all farms [96].

Like in human medicine, machine learning models for animal diseases should gradually form an evaluation pathway of “external validation—continuous monitoring—randomized controlled trials (RCTs)/prospective trials” as they transition into real-world deployment [97]. However, in the context of animal viral diseases and livestock production practices, models for key diseases such as African swine fever, Newcastle disease, foot-and-mouth disease, or avian influenza still lack systematic external validation and prospective trials conducted in real farm environments. Furthermore, randomized field trials that link model performance to farming outcomes and economic benefits are largely absent. While a small number of machine learning-based early-warning systems using farm big data have been attempted in actual farm settings, much of the work remains at the “offline retrospective evaluation” stage and has not yet formed a mature evidence-based framework comparable to that seen in human medicine.

In summary, from cross-species generalizability and distribution drift due to viral evolution to the lack of external validation and prospective evaluation in real production environments, the application of machine learning and deep learning in animal viral disease control still faces multiple practical challenges. Combining earlier case studies of African swine fever protein function prediction, Newcastle disease multimodal diagnostics, and foot-and-mouth disease and avian influenza risk assessment, the relevant methodologies can currently be viewed only as upstream research tools for accelerating candidate mechanism screening and hypothesis generation rather than as mature decision-support systems ready for use in large-scale farming scenarios. Only when significant progress is made in cross-species data accumulation, dynamic model updates, and external validation and prospective trials based on real farm environments can machine learning and deep learning provide more stable and sustainable support in the practice of animal viral disease control.

## 8. Conclusions and Discussion

From early-stage sequence alignment and statistical analysis to the current use of complex deep neural networks and graph-based models, computational methodologies have continually injected new vitality into virological research. In protein–protein interaction (PPI) prediction, DL models now achieve high accuracy, providing valuable leads for experimental validation and antiviral target discovery. In drug development, virtual screening techniques that integrate molecular docking, QSAR modeling, and DL have greatly improved the efficiency of candidate selection [98]. In vaccine design, ML-based epitope prediction algorithms can accurately identify potential immunogenic sites, offering strong theoretical support for antigen design [99]. In addition to the technical and application-level challenges discussed above, emerging large language models (LLMs) are likely to shape future directions in animal infectious disease management. Owing to their strong capabilities in terms of knowledge integration and natural-language reasoning, LLMs can rapidly synthesize dispersed scientific evidence, assist in hypothesis generation, and provide decision support for veterinarians and researchers. When adapted to domain-specific corpora, LLMs may also extract structured information from unstandardized farm records, diagnostic reports, and clinical notes, thereby improving surveillance efficiency and situational awareness. Furthermore, multimodal LLMs have the potential to link text, genomic sequences, sensor-derived signals and epidemiological metadata into unified analytical frameworks, enabling more holistic risk assessment. As these models become more transparent, reliable, and aligned with veterinary needs, LLM-based AI assistants could serve as valuable complementary tools to existing ML pipelines, supporting early detection, targeted intervention, and coordinated disease control strategies.

Multiple epitope vaccine strategies, which combine computational prediction with laboratory verification, show considerable promise for the prevention of animal viral diseases. Similarly, in viral evolution and biomarker discovery, ML has proven instrumental in tracking evolutionary trajectories and predicting mutation trends [100]. Moreover, the use of multiomics data for biomarker identification is opening new avenues for early diagnosis and precision medicine in veterinary science [101]. In practical applications, the integration of deep learning with machine learning has generated synergistic effects in the study of animal infectious diseases, and this combined approach has already led to breakthroughs in several domains. For example, in the diagnosis of African swine fever, Miller and colleagues developed a rapid, sensitive, and user-friendly field-deployable diagnostic tool based on deep learning; by improving the accuracy of lateral-flow assay (LFA) strip image classification, their system provided substantial support for the onsite detection of infected pigs [102]. In the area of viral detection, the deep learning-based image recognition framework proposed by Vadivu and Neethirajan (2024) achieved early identification of bovine cutaneous viral diseases such as lumpy skin disease, thereby offering effective technological aid for livestock pathogen surveillance [103]. Moreover, Halev and coworkers (2023) applied machine-learning models to predict day-to-day outbreak risks in swine herds—including those involving viral respiratory and enteric pathogens—and demonstrated the considerable potential of AI for early warning in pig production systems, providing valuable technical support for outbreak prevention and control [72] in Table 1.

Despite the substantial promise of machine learning and deep learning in research on viral diseases of livestock, the technological and practical challenges that surround their application remain impossible to overlook. Issues ranging from data quality, model interpretability, and computational demands to viral evolution, data integration, and ethical constraints all impose strict requirements on researchers working toward real-world deployment. To address these challenges, future work should concentrate on several key directions: first, the construction of high-quality validation datasets—particularly standardized, cross-species and cross-regional datasets—and their controlled sharing to increase the reliability and generalizability of model training. Second, research on model interpretability has advanced, especially in contexts related to clinical decision-making and public health applications, with a focus on developing deep learning approaches that offer greater transparency and operational trust. Finally, strengthening the integration of multiomics data with PPI-level validation will be critical; the potential of machine learning and deep learning for data fusion is considerable, especially when genomic, transcriptomic, and proteomic information is analyzed jointly to support more accurate prediction of viral evolution and the identification of vaccine-candidate targets.

In conclusion, while ML and DL have demonstrated immense potential in animal disease research, realizing their full value requires overcoming technical, methodological, and ethical challenges. Through sustained innovation, high-quality data generation, interdisciplinary cooperation, and experimental confirmation, this field is poised for major breakthroughs in the coming years. These advancements will not only strengthen the prevention and control of African swine fever and other viral diseases but also enhance global food security, public health resilience, and the sustainable development of animal husbandry.

## Figures and Tables

**Table 1:** Scientific questions in animal viral disease management that can be addressed via machine learning and deep learning.

Scientific Question in Animal Viral Disease Management	How ML/DL Contributes	Typical Data/Examples	Representative References (No.)
Sequence-based surveillance and molecular diagnostics	Automates lineage, variant and pathogenicity labeling from viral sequences; replaces manual phylogeny and hemagglutination tests; rapidly flags high-risk strains.	PRRSV ORF5 lineage/variant classification; deep learning on AIV HA sequences/structures for H5/H7 pathogenicity; ML prediction of NDV antigenic distance from F/HN gene features.	[19,20,21,22,23,24,25]
Sensor- and image-based diagnostics and monitoring	Uses CNNs and other DL models on audio, RGB and thermal signals for noninvasive health monitoring and flock-level early warning; supports low-cost, on-farm screening.	Poultry vocalization recordings for 1D-CNN health-status classification; CNN analysis of broiler feces images (coccidiosis, salmonellosis, ND); thermography + SVM/ANN for early detection of avian influenza and ND.	[26,27,28,29,30]
Epidemiological modeling and risk assessment	Learns associations between outbreaks and risk factors; builds spatiotemporal prediction and farm-level risk-scoring models to support targeted surveillance and control.	Farm-level FMD datasets for RF/CHAID outbreak prediction; PRRSV biosecurity-variable datasets; BVDV and animal-welfare inspection data used to derive risk scores and priority farm lists.	[31,32,33,34,35]
Viral mechanisms and host–virus molecular interactions	Predict viral protein functions and annotate unknown ORFs; infer virus–host PPIs; use structure prediction and neural-network PPI models to map key domains and network hubs.	ASFV multigene families and ORFs; ASFV–host PPI networks; AlphaFold-based ASFV structures (e.g., CD2v, Georgia 2007 proteome); generic virus–host PPI datasets and benchmarks.	[36,37,38,39,40,41,42,43,44,45,46,47,48,49]
Biomarker discovery and multiomics integration	Applies ML/DL to integrate genomics, transcriptomics, proteomics and metabolomics; identifies diagnostic/prognostic biomarkers; defines molecular subtypes and mechanistic pathways.	Multiomics datasets for H9N2 in laying hens; supervised and multitask ML frameworks (SVM, RF, XGBoost) for biomarker selection; AE/VAE/GNN-based integration; PRRS metabolomic/lipidomic biomarkers; BRD and swine influenza multiomics case studies.	[50,51,52,53,54,55,56,57,58,59]
Antiviral target prediction and drug discovery (network pharmacology + ML)	Combines network pharmacology with ML/DL (QSAR, docking-driven ML, DTI prediction, graph models) to prioritize antiviral targets, rank compounds and support multitarget drug strategies.	ASFV polymerase and other viral targets; large virtual compound libraries; compound–target affinity and DTI datasets; microRNA–mRNA regulatory networks for target discovery.	[60,61,62,63,64,65]
Vaccine antigen and epitope prediction	Supports reverse vaccinology and immunoinformatics pipelines; predicts protective antigens and B-/T-cell epitopes; assists multiepitope vaccine design and in silico immune simulation.	Viral genomes and proteomes (ASFV, other pathogens); epitope databases; AlphaFold structural models; RV frameworks (e.g., Vaxign-DL); multiepitope ASFV vaccine design (BepiPred-2.0, AlphaFold, C-ImmSim); DL-designed SARS-CoV-2 vaccine subunits.	[66,67,68,69,70,71]
Farm-level early warning and outbreak prediction	Integrates longitudinal production, health and environmental data to predict short-term infection risk at herd level and flag high-risk farms for intervention.	Time series from swine production systems (mortality, performance, management, environment) used by ML models for daily outbreak-risk prediction in herds.	[72]

**Table 2:** Representative ML/DL case studies in animal viral disease management.

Representative ML/DL Case Study	Primary Data Type(s)	ML/DL Approach and Scientific Question Addressed	References (No.)
PRRSV ORF5 lineage and “genetic variant” classification	PRRSV ORF5 amino acid sequences; physicochemical features; large field-surveillance datasets.	Supervised ML (e.g., random forests) classifies ORF5 sequences into lineages/”genetic variants”, replacing manual alignment and phylogeny in routine sequence-based surveillance.	[19,20,21]
Pathogenicity prediction of avian influenza and antigenic distance estimation for NDV	AIV genomes/HA sequences and predicted HA 3D structures; NDV F/HN sequence features.	DL on HA structural representations distinguishes highly pathogenic H5/H7 from low-pathogenic AIV; RF/SVM on F/HN features predict NDV antigenic distance for risk assessment and vaccine-strain selection.	[22,23,24,25]
Audio-, image-, and thermography-based health monitoring in poultry	Barn audio; RGB images of broiler droppings; infrared thermograms from experimental infection.	1D-CNNs classify flock health from vocalizations; CNNs detect coccidiosis, salmonellosis and ND from fecal images; SVM/ANN on thermal features enable noninvasive early detection of AIV and ND.	[26,27,28,29,30]
Foot-and-mouth disease (FMD) outbreak prediction at farm level	Farm-level surveillance and management data; herd size, vaccination and local context variables.	Decision trees, RF, CHAID and related ML classifiers predict whether cattle farms will experience FMD outbreaks, providing operational early-warning tools for endemic settings.	[31,32,73]
Farm-level risk scoring and biosecurity analysis for PRRSV and BVDV	Biosecurity, management, animal-movement and demographic data with PRRSV/BVDV status.	RF and related ML methods rank biosecurity factors, predict farm-level risk and support data-driven biosecurity recommendations and risk-based inspection strategies.	[33,34,35]
ASFV protein function annotation and structure-based mechanistic inference	ASFV genomes and protein sequences; virus–host PPI networks; AlphaFold-predicted ASFV structures.	Multitask ML improves functional annotation of ASFV proteins and poorly characterized ORFs; PPI network analysis identifies key viral/host nodes; DL-based structure prediction links domains/residues to hemadsorption and virulence.	[36,37,38,39,40,41,42]
Multiomics biomarker discovery and mechanistic dissection (H9N2, PRRS, BRD, swine influenza)	Genomic, transcriptomic, proteomic, metabolomic and lipidomic profiles from infected vs. healthy animals or different disease phenotypes.	ML (e.g., XGBoost, RF, SVM) identifies H9N2 biomarkers in laying hens; PRRS metabolomics/lipidomics highlight lysophosphatidic acid; multiomics ML frameworks for BRD and swine influenza reveal susceptibility loci, pathways and early host-response signatures.	[56,57,58,59]
Network pharmacology plus ML for ASFV antiviral discovery	Large compound libraries; docking/affinity features; ASFV polymerase and other viral targets; DTI and regulatory networks.	ML models combined with molecular docking rapidly rank compounds and identify ASFV polymerase inhibitors; DL/ML (GCN, VAE, RF, SVM) predict drug–target interactions and support host-directed and polypharmacology antiviral strategies.	[60,61,62,63,64,65]
ML/DL-assisted reverse vaccinology and epitope-based vaccine design	Viral genomes/proteomes; known/predicted B- and T-cell epitopes; structural models; in silico immune-simulation outputs.	ML/DL RV pipelines predict protective antigens; RF/SVM/DL epitope predictors select linear and conformational epitopes; integrated platforms (epitope prediction + AlphaFold + immune simulation) design multiepitope ASFV vaccines and DL-derived SARS-CoV-2 vaccine subunits.	[66,67,68,69,70,71]

## Data Availability

No new data were created or analyzed in this study. Data sharing is not applicable to this article.
